# Retrobulbar Blood Flow Parameters in Patients With Anisometropic Amblyopia

**DOI:** 10.7759/cureus.35025

**Published:** 2023-02-15

**Authors:** Emre Aydın, Eren Özgür, Aykut İnsan, Enes Gürün

**Affiliations:** 1 Ophthalmology, Rize Şar Hospital, Rize, TUR; 2 Radiology, Istanbul Education and Research Hospital, Istanbul, TUR; 3 Radiology, Osmaniye State Hospital, Osmaniye, TUR; 4 Radiology, Samsun University, Samsun, TUR

**Keywords:** retrobulbar, ocular blood flow, color doppler ultrasonography, vision, anisometropic amblyopia

## Abstract

Introduction

We aim to compare retrobulbar blood flow parameters between the amblyopic eye and the fellow eye in patients with anisometropic amblyopia.

Methods

Peak systolic velocity (PSV) and end-diastolic velocity (EDV) of the ophthalmic artery (OA), central retinal artery (CRA), and posterior ciliary artery (PCA) were measured by color Doppler imaging (CDI), and the resistivity index (RI) and pulsatility index (PI) were calculated in 62 patients aged 12-40 years with anisometropic amblyopia.

Results

The mean PSV values of OA, CRA, and PCA in amblyopic and fellow eyes were 30.38 ± 10.34, 8.45 ± 2.27, and 8.03 ± 2.77, and 33.73 ± 14.46, 8.35 ± 2.05, and 8.81 ± 2.77, respectively. The mean EDV values of OA, CRA, and PCA in amblyopic and fellow eyes were 6.86 ± 2.64, 1.47 ± 1.59, and 1.94 ± 2.03, and 8.57 ± 4.30, 1.80 ± 1.73, and 2.32 ± 1.20, respectively. The mean RI values of OA, CRA, and PCA in amblyopic and fellow eyes were 0.77 ± 0.10, 0.85 ± 0.14, and 0.78 ± 0.15, and 0.75 ± 0.07, 0.79 ± 0.20, and 0.74 ± 0.13, respectively. OA-PSV and OA-EDV values were significantly lower in the amblyopic eye than in the healthy eye (p < 0.05). OA-RI values were significantly higher (p < 0.05) in the amblyopic eye than in the healthy eye.

Conclusions

Considering the decrease in PSV and EDV and the increase in RI, which are the blood flow parameters of the amblyopic eye, our study may provide guidance to focus on increasing blood flow in the treatment of amblyopia.

## Introduction

Amblyopia is a developmental visual system disorder characterized by a decrease in best-corrected visual acuity (BCVA) in an eye without organic pathology. It usually develops in the sensitive period of childhood from birth to age six, in the case of physical or physiological abnormal visual input to one of the eyes [1]. Also, the development of orbital structures also varies according to age [2].

There are three main types of amblyopia: anisometropia, strabismus, and deprivation. Anisometropic amblyopia usually occurs in children with refractive error between the eyes due to hyperopia or astigmatism, and it occurs in the eye with higher refractive error [3]. According to Weakley, there is an increased risk of amblyopia in those with >2 diopters (D) myopic anisometropia, >1 D hyperopic anisometropia, and >1.5 D astigmatic anisometropia [4]. The American Academy of Ophthalmology in the “Preferred Practice Model” recommends that anisometropic patients with ≥1.5 D hyperopia, ≥2.0 D myopia, or ≥2.0 D astigmatism be followed up for the development of amblyopia [5].

In this study, we aimed to record and compare the retrobulbar blood flow parameters between the amblyopic eye and the healthy eye of patients with anisometropic amblyopia aged >12 years who have passed the sensitive period for amblyopia.

This article was previously posted to the Research Square preprint server on May 10, 2022.

## Materials and methods

The study was approved by Ağrı İbrahim Çeçen University Ağrı Training and Research Hospital Ethics Committee (00159911404). All patients received full verbal and written details of the study and provided informed written consent before enrollment. All steps of the study were planned and continued according to the principles outlined in the Declaration of Helsinki. The healthy eye with full visual acuity and the amblyopic eye of 62 volunteer patients aged 12-40 years without ocular and/or systemic disease were included in the study between January 2021 and January 2022.

Clinically, anisometric amblyopia is defined as a difference of 0.20 logMAR in BCVA between the two eyes, and we included those with at least 0.20 logMAR difference in visual acuity between the two eyes in the amblyopic patient group [6]. In the ocular examination, a standard ophthalmological examination was performed, including evaluation of eye alignment with the distant and near on-off tests and the alternating closure test, measurement of corrected amblyopic logMAR visual acuity, measurement of refractive error after providing adequate pupil dilation by instilling one drop of 1% cyclopentolate two times at five-minute intervals, anterior segment examination, fundus examination with slit-lamp biomicroscopy, and intraocular pressure (IOP) measurement with applanation tonometry. Cycloplegic refractive error was measured with an autorefractor (Topcon KR 8900, Tokyo, Japan), followed by verification of autorefractor measurements by an experienced retinoscopist and, when possible, by a subjective refinement in subjects under cycloplegia. The BCVA was assessed using a large-format standard light box (ESV3000 with LED light, Vector Vision, Greenville, OH, USA) positioned 4 m from the patient.

Patients with a history of amblyopia due to strabismus and deprivation, a history of intraocular surgery, a history of glaucoma, a history of laser surgery, a history of cataract, corneal pathologies such as corneal scarring, a history of contact lens use, and a history of topical and systemic drug use and patients with retinal and optic disc disorders, carotid artery pathology on carotid Doppler imaging, ocular trauma or structural ocular abnormalities, including neurological disorders that may affect visual acuity, and a BCVA of less than 40/40 in the healthy eye were excluded from the study.

**Figure 1:**
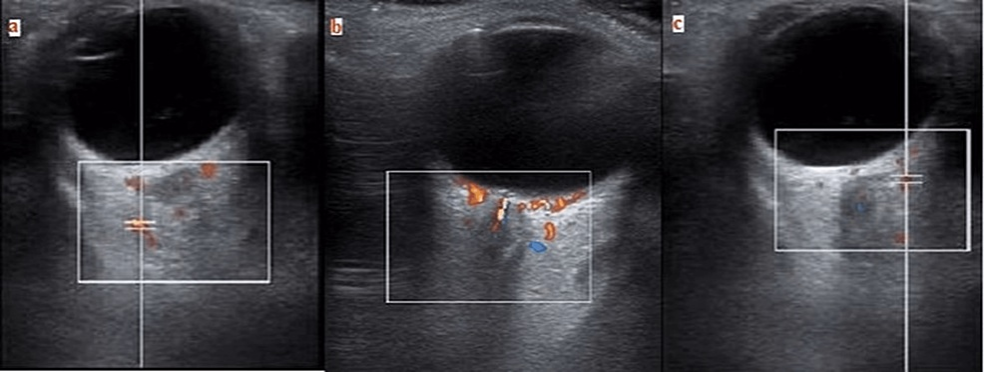
Measuring technique of ophthalmic artery (a), central retinal artery (b), and posterior ciliary artery (c) with orbital Doppler

To minimize the effects of diurnal variation, all retrobulbar color Doppler imaging (CDI) studies were recorded at the same time of day (between 9:00 am and 11:00 am) by the same sonographer for each subject. A color Doppler imaging device (Toshiba Aplio MX ultrasound system, Toshiba Medical Systems, Tustin, California) with an 11-MHz linear phased-array transducer was used to evaluate blood flow velocity. Patients were in a supine position with their heads leaned forward at about a 30-degree angle and their eyes closed during the examination. The surface of the transducer probe was coated with hydroxyethylcellulose, an acoustic gel. Acoustic signals from the central retinal artery (CRA), posterior ciliary artery (PCA), and ophthalmic artery (OA) were detected by placing the probe on the eyelid without applying pressure and gently moving it over the eyelid. Initially, a color Doppler image was obtained at the level of the optic nerve (ON), which is the most useful location for identifying retrobulbar vessels. CRA measurements were taken at the optic nerve head level. OA flow measurements were made approximately 10-15 mm behind the globe, from which stronger ultrasound signals were received. PCA images were obtained from the temporal and nasal regions of the optic nerve located at the posterior pole of the eye (Figure 1a-1c).

The angle between the transducer and vessel direction was optimally adjusted.

Peak systolic velocity (PSV) and end-diastolic velocity (EDV) were measured, and the resistance index (RI) and pulsatility index (Pourcelot index) (PI) were computed. The RI and PI are typically used to assess the resistance in the vascular system. The RI is a flow parameter calculated with the formula (PSV-EDV)/(PSV) using the maximum-minimum rate values during a cardiac cycle. The PI, also known as the gosling index, is calculated automatically by the ultrasound device with the maximum-minimum velocity values obtained from the Doppler shift frequency during the cardiac cycle.

The Statistical Package for Social Sciences (SPSS) version 27.0 (IBM SPSS Statistics, Armonk, NY, USA) was used in the analysis. In the descriptive statistics of the data, mean, standard deviation, median lowest, median highest, frequency, and ratio values were used. The distribution of variables was measured using the Kolmogorov-Smirnov test. The Wilcoxon test was used in the analysis of dependent quantitative data. A p value of less than 0.05 was considered to be statistically significant.

## Results

The amblyopic and intact eyes of 62 patients (36 females and 26 males) with anisotropic amblyopia were included in this study. The mean age was 25.9 ± 7.418 years (12-40 years). There were 28 right and 34 left amblyopic eyes (Table 1).

**Table 1:** Demographics and characteristics of the patients SD: standard deviation

		Minimum-maximum			Median	Mean ± SD/number (%)
Age		12	-	40	25.00	25.29 ± 7.418
Gender	Female					36 (58.1%)
	Male					26 (41.9%)

Visual acuity was 1.0 in the healthy eye and 0.45 ± 0.22 in the amblyopic eye. The mean PSV values of OA, CRA, and PCA in amblyopic and fellow eyes were 30.38 ± 10.34, 8.45 ± 2.27, and 8.03 ± 2.77, and 33.73 ± 14.46, 8.35 ± 2.05, and 8.81 ± 2.77, respectively. The mean EDV values of OA, CRA, and PCA in amblyopic and fellow eyes were 6.86 ± 2.64, 1.47 ± 1.59, and 1.94 ± 2.03, and 8.57 ± 4.30, 1.80 ± 1.73, and 2.32 ± 1.20, respectively. The mean RI values of OA, CRA, and PCA in amblyopic and fellow eyes were 0.77 ± 0.10, 0.85 ± 0.14, and 0.78 ± 0.15, and 0.75 ± 0.07, 0.79 ± 0.20, and 0.74 ± 0.13, respectively. The mean PI values of OA, CRA, and PCA in amblyopic and fellow eyes were 1.73 ± 0.62, 2.57 ± 1.30, and 1.88 ± 0.99, and 1.65 ± 0.39, 2.32 ± 1.27, and 1.76 ± 0.67, respectively.

**Figure 2:**
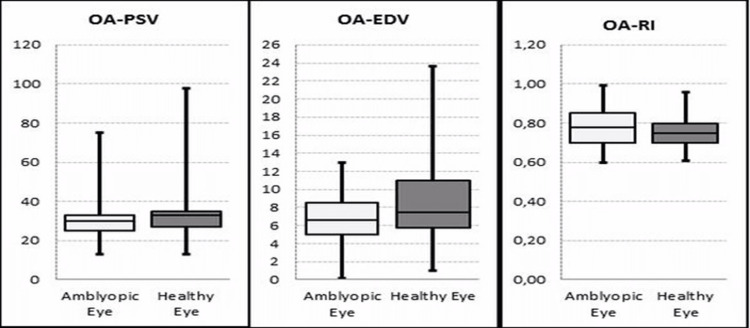
Boxplot of PSV, EDV, and RI of OA in patients’ amblyopic eye and healthy eye PSV: peak systolic velocity, EDV: end-diastolic velocity, RI: resistivity index, OA: ophthalmic artery

Visual acuity in the amblyopic eye was significantly lower than in the healthy eye (p < 0.05). The OA-PSV and OA-EDV values in the amblyopic eye were significantly lower (p < 0.05) than in the healthy eye. OA-RI values were significantly higher (p < 0.05) in the amblyopic eye than in the healthy eye (Figure 2).

In OA-PI, CRA-PSV, CRA-EDV, CRA-EDV, CRA-PI, PCA-PSV, PCA-EDV, PCA-PI, PCA-RI, and intraocular pressure value parameters, there was no statistically significant difference between the amblyopic and healthy eye (Table 2).

**Table 2:** Comparison of CDI parameters, visual acuity, and IOP between amblyopic and healthy eye CDI: color Doppler imaging, OA: ophthalmic artery, CRA: central retinal artery, PCA: posterior ciliary artery, PSV: peak systolic velocity, EDV: end-diastolic velocity, RI: resistivity index, PI: pulsatility index, IOP: intraocular pressure, SD: standard deviation

	Amblyopic eye				Healthy eye				p	
	Mean ± SD			Median	Mean ± SD			Median		
Visual acuity	0.45	±	0.22	0.50	1.00	±	0.00	1.00	0.001
OA-PSV	30.38	±	10.34	30.00	33.73	±	14.46	33.00	0.010
OA-EDV	6.86	±	2.64	6.60	8.57	±	4.30	7.50	0.001
OA-PI	1.73	±	0.62	1.65	1.65	±	0.39	1.62	0.109
OA-RI	0.77	±	0.10	0.78	0.75	±	0.07	0.75	0.021
CRA-PSV	8.45	±	2.27	8.00	8.35	±	2.05	8.00	0.483
CRA-EDV	1.47	±	1.59	0.80	1.80	±	1.73	1.60	0.910
CRA-PI	2.57	±	1.30	2.20	2.32	±	1.27	2.06	0.263
CRA-RI	0.85	±	0.14	0.90	0.79	±	0.20	0.82	0.453
PCA-PSV	8.03	±	2.77	7.80	8.81	±	2.77	8.20	0.200
PCA-EDV	1.94	±	2.03	1.70	2.32	±	1.20	2.40	0.115
PCA-PI	1.88	±	0.99	1.50	1.76	±	0.67	1.67	0.719
PCA-RI	0.78	±	0.15	0.76	0.74	±	0.13	0.75	0.173
IOP	14.39	±	3.06	14.00	14.48	±	2.57	15.00	0.917

## Discussion

Color Doppler imaging (CDI) is a safe, noninvasive, and repeatable method used for the localization of orbital vessels and assessment of blood flow velocities. It is increasingly used in the evaluation of many abnormalities affecting orbital hemodynamics [7]. CDI can provide information about retrobulbar vessels such as OA, CRA, and PCA [8]. Although blood volume cannot be measured with spectral Doppler, quantitative velocity measurements and resistance indices can be calculated, and since there is a good correlation between blood velocities and blood flow, this is not considered a deficiency [9]. In addition, CDI has been found to be useful in investigating changes in retrobulbar blood flow after some ophthalmic surgeries such as scleral buckling, trabeculectomy, and strabismus surgery [10,11].

In anisometropia, an unequal refractive error between the two eyes creates abnormal binocular interaction and/or visual deprivation. Patients with anisometropic amblyopia usually do not have an identifiable ocular defect, and the visual acuity of the healthy eye is normal, which makes diagnosis difficult and delays treatment [12,13]. In anisotropic amblyopia, different stimuli from the two eyes cause deficiencies in visual processing in the primary visual cortex. The imbalance in image quality between the two eyes can greatly reduce or even completely destroy stereo vision (3D vision) [14,15]. In addition to decreased contrast sensitivity in amblyopic patients, deficiencies in higher-order functional functions such as motion detection, temporal integration, peripheral vision, and the ability to perceive more than one element are also detected [16,17]. Higher-grade deficits in eye-hand motor coordination and global movement processing have also been reported [18]. Binocular experience is thought to play an important role in the formation of these disorders. These findings suggest that a new treatment approach designed to treat binocular dysfunction as the primary deficiency in amblyopia may be needed [19].

In the study in which patients with axial anisometric and long axial length (AL) were evaluated with CDI, it was found that the pulsatile ocular blood flow (POBF) and blood flow velocity of OA were decreased, but it was not statistically significant [18]. Lempert discovered that the optic disks of hypermetropic eyes with strabismus (with and without amblyopia) were disproportionately small in size compared to hypermetropic eyes without amblyopia or esotropia in photos of the optic nerves (ON) of human eyes with amblyopia [19,20]. He theorized that amblyopia visual impairment is linked to ON hypoplasia with relative microphthalmos. Rasch et al. also observed internal plexiform layer thinning and nucleolar volume decrease in the ganglion cell cytoplasm, as well as a reduction in ON size in amblyopic eyes, in an experimental investigation [21]. In another investigation, scanning laser polarimetry was used on patients with unilateral strabismic or anisometropic amblyopia. Between the amblyopic and fellow eyes, there was no statistically significant difference in retinal nerve fiber layer (RNFL) thickness [22].

There was no statistically significant difference in retrobulbar blood flow between the amblyopic eye and the healthy eye in patients with anisometropic amblyopia [23]. The larger number of patients in our study is more significant in terms of literature. In our study, in the comparison of the amblyopic eye and the healthy eye of patients diagnosed with anisometropic amblyopia, retrobulbar blood flow parameters PSV of OA and EDV of OA were found to be decreased in the amblyopic eye compared to the healthy eye, and it was found to be statistically significant. In addition, the RI value of OA was discovered to be higher in the amblyopic eye compared to the healthy eye, and it was determined to be statistically significant. Although the PI value of OA increases, it is not statistically significant. Visual acuity was also decreased in the amblyopic eye, which was statistically significant. OA is the main feeding vessel of the eye and then divides into PCA and CRA branches. CRA’s EDV was reduced, but there was no statistically significant difference. Although CRA’s RI and PI values increased, there was no statistically significant difference. Despite the fact that PCA’s PSV and EDV were reduced, there was no statistically significant difference. Despite the fact that PCA’s RI and PI increased, there was no statistically significant difference.

Considering the decrease in PSV and EDV, and the increase in RI, which are the blood flow parameters of the amblyopic eye, our study may provide guidance to focus on increasing blood flow in the treatment of amblyopia. For example, following exercise, the mean ocular perfusion pressure increased considerably. Both the ophthalmic and central retinal artery study groups found all of these effects [24]. According to Okuno et al., choroid-retina blood flow was reduced by 6% in measurements taken 60 minutes after the oral administration of caffeine [25]. Caffeine may raise blood vessel resistance and decrease blood flow in the optic nerve head and choroid-retina circulation, according to the researchers. Several pharmacological investigations have looked into the effects of various medicines on retrobulbar hemodynamics [26,27].

While there is substantial evidence for increased ocular blood flow during dorzolamide treatment, it is unclear to what extent is this enhanced ocular blood flow related to IOP reduction alone or to additional pharmacological effects on the vascular system. In addition, an increase in blood velocities following topical dorzolamide has been described in the literature [28]. In their investigation, Källberg et al. discovered that systemically administered amlodipine enhances CDI-determined blood flow velocities and decreases vascular resistance index in normal dogs with OA and PCA [29]. According to the results of these studies, treatments such as limiting coffee consumption in patients with amblyopia, encouraging exercise, and using retrobulbar blood flow-increasing treatments such as diazomid and amlodipine may be among the recommended methods for the treatment of amblyopia in the future.

Our study’s limitations include the large age range (12-40 years). However, by utilizing the fellow eye as a control, we were able to rule out the effects of age, systemic disease, and other factors on ocular blood flow.

## Conclusions

In our study, visual acuity, and OA-PSV and OA-EDV values in the amblyopic eye were significantly lower than in the healthy eye, while OA-RI values were found to be significantly higher in the amblyopic eye than in the healthy eye. Considering the decrease in PSV and EDV, and the increase in RI, which are the blood flow parameters of the amblyopic eye, our study may provide guidance to focus on increasing blood flow in the treatment of amblyopia.
